# Nafion-Protected Sputtered-Bismuth Screen-Printed Electrode for On-site Voltammetric Measurements of Cd(II) and Pb(II) in Natural Water Samples

**DOI:** 10.3390/s19020279

**Published:** 2019-01-11

**Authors:** Samuel Frutos-Puerto, Conrado Miró, Eduardo Pinilla-Gil

**Affiliations:** 1 Department of Analytical Chemistry, University of Extremadura, Av. de Elvas, s/n, 06006 Badajoz, Spain; samfrutosp@unex.es; 2 Department of Applied Physics, University of Extremadura, Av. de la Universidad, s/n, 10005 Cáceres, Spain; cmiro@unex.es

**Keywords:** cadmium, lead, nafion, bismuth-sputtered screen-printed electrode, SWASV

## Abstract

In this work, we explore the protection with Nafion of commercial sputtered-bismuth screen-printed electrodes (Bi_SP_SPEs), to improve its ability for on-site determination of Cd(II) and Pb(II) ions in ambient water samples. The modified screen-printed platform was coupled with a miniaturized cell, in combination with a battery-operated stirring system and a portable potentiostat operated by a laptop for decentralized electrochemical measurements using Square-Wave Anodic Stripping Voltammetry (SWASV). We also describe a detailed electrode surface characterization by microscopy and surface analysis techniques, before and after the modification with Nafion, to get insight about modification effect on signal size and stability. Optimization of the chemical composition of the medium including the optimization of pH, and instrumental parameters, resulted in a method with detection limits in the low ng/mL range (3.62 and 3.83 ng·mL^−1^ for Cd and Pb respectively). Our results show an improvement of the sensitivity and stability for Nafion-protected Bi_SP_SPEs in pH = 4.4 medium, and similar or lower detection limits than comparable methods on commercial Bi_SP_SPEs. The values obtained for Pb(II) and Cd(II) in natural water samples agreed well with those obtained by the much more costly Inductively Coupled Plasma Mass Spectrometry, ICP-MS, technique as a reference method (recoveries from 75% to 111%).

## 1. Introduction

Cd(II) and Pb(II) are well-known contaminants and potentially toxic elements that may be present in drinking water, groundwater, and soil. Anthropogenic activities, such as mining, industrial processing, vehicle emissions or agricultural fertilization, may lead to increase the concentration of this metals that may enter the human body through drinking water, air, food, or by absorption through the skin. The use of bismuth as a working electrode for the electrochemical determination of these trace elements in environmental samples have been largely proved as an effective alternative instead of mercury drop working electrodes [1,2,3,4,5,6]. These bismuth-based electrodes studies are focused in solving some of the problems that mercury electrodes show, namely, technical constrictions or toxicity. Among the possibilities offered by the use of bismuth as electrode material [7], bismuth film on conventional electrodes (mostly glassy carbon) can be considered the most consolidated approach to this aim [8]. However, while this method shows good results, Bi-coated screen printed electrodes (SPE) have been gaining importance [9,10]. Taking this trend into account, it is widely known that SPEs have significant advantages, including low cost, small size, commercial availability and, as the most relevant aspect for decentralized analysis, the possibility of being connected to a portable measurement system [11,12,13], making it suitable for on-site and even in-situ determinations.

SPE’s are usually constructed with a three-electrode configuration (working electrode, pseudo-reference electrode and auxiliary electrode) and are commercially available, with a wide range of inks containing the desired material for the working electrode.

An alternative to commercial SPEs is coating the substrate with bismuth by three general methods: Ex-situ plating (preplated) method (electroplated Bi on the electrode substrate surface prior the analysis) [5,14], in-situ plating method (Bi added at the sample during the analysis) [15,16], bulk method: Mix the graphite ink with a bismuth precursor during the SPE production [2].

Another option, apart from these general methods, is to sputter the bismuth on a ceramic substrate leading to a Bi_SP_SPE. The applicability of this electrode has been demonstrated for stripping analysis as an advantageous technique for the analysis of trace metals of environmental concern [17,18,19]. Is important to note that, in the case of Bi_SP_SPE, some of the published studies employ it in a conventional electrochemical cell with external reference and counter electrodes and only taking the advantages of the working electrode with impairment in the portable features [18,19].

One step further in the use of SPEs is modifying the surface of the working electrode by coating it with colloidal nanoparticles or additives in order to improve the electrode response (selectivity and sensitivity) or to protect its surface (stability) against colloidal particles presents in the sample and that may deteriorate it during the analysis. Within this wide variety of additives, we can highlight Nafion [20,21], EDTA [22,23] or Chitosan [24].

Nafion is a sulfonated cation-exchange polymer, and it has both favorable conductive matrix and anion sites that usually is employed with carbonaceous materials, such as nanofibers [25,26] or nanotubes [20,27] along with developed methods, such as linear sweep voltammetry, differential pulse voltammetry and square wave voltammetry. Mandil and Amine [28] described the use of Nafion to cover Bi-film preplated on SPEs for the determination of Cd and Pb in ceramic leachates. Kokkinos et al. [29] studied the analytical utility of Nafion in microfabricated bismuth film electrodes for the determination of Pb(II) and Cd(II) by anodic stripping voltammetry and Jing Li et al. [30] employed an in situ plated bismuth film electrode joint with graphene nanosheets dispersed in Nafion.

In this work, we have aimed to test the analytical improvements of Nafion protection on commercial Bi_SP_SPE, specifically the effect of the polymeric cover on the conservation of the original 3D superficial structure of the electrode. We have applied the modified electrode to measure Pb(II) and Cd(II) in natural water samples (certified reference materials and real samples) by Square-Wave Anodic Stripping Voltammetry. Our study includes a detailed exploration of the evolution of the Nafion-protected surface during the measurements, performed by Scanning Electron Microscopy (SEM). Finally, we evaluate the portable features of the overall measurement system.

## 2. Materials and Methods

### 2.1. Chemicals and Reagents

The Cd(II) and Pb(II) standard stock solutions (1000 mg·L^−1^) were supplied by Sharlab (Barcelona, Spain) and diluted as required. All solutions were prepared from ultra-pure water (18.2 MΩ·cm) obtained from a Wasserlab Ultramatic system (Navarra de Tratamiento de Agua S.L., Pamplona, Spain). Hyperpure grade HCl, supplied by Panreac (Barcelona, Spain), was used to prepare a 10^−2^ M solution that was used to adjust the samples to pH 2. Acetic/acetate buffer solution (10^−2^ M) made from HPLC grade acetic acid (Panreac) and sodium acetate (Merck, Darmstadt, Germany) was used for pH 4.5 and pH 5.5 adjustment of the multielement solutions. All the material used was properly washed by immersion in a 10% sub-boiled HNO_3_ solution for one week. The sub-boiled HNO_3_ was obtained from a quartz sub-boiling system (Kürner, Rosenheim, Germany). All the measurements were carried out after adjusting Cl^−^ to 4·10^−2^ M by KCl (Chem-Lab, Fisher, UK), for better performance of the Ag strip pseudoreference electrode. To protect the working electrode, an ethanol-diluted nafion perfluorinated solution (Fluka, St. Louis, MO, USA), was added to the surface.

### 2.2. Apparatus

**Figure 1:**
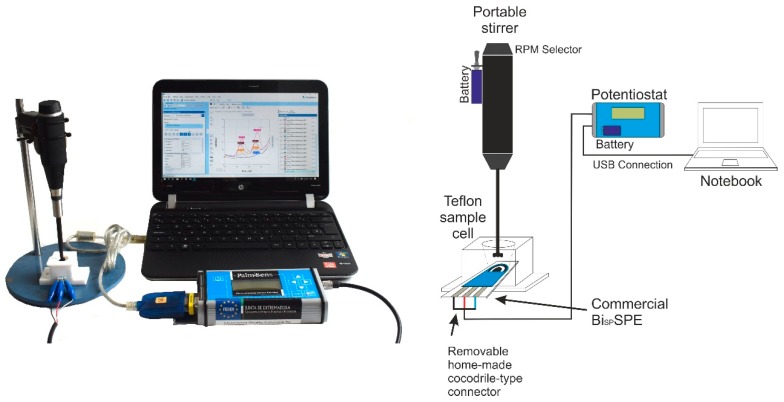
Schematic of the experimental setup for the on-site determination of Cd(II) and Pb(II) by Bi_SP_SPE.

A PalmSens potentiostat/galvanostat (Palm Instruments BV, The Netherlands) was used to carry out the voltammetric measurements. This instrument is controlled by PSTrace v.5.4 software provided by PalmSens and connected to a Notebook PC. The measurement set also included a battery operated handy-modified manually-controlled stirrer (Radiometer, Copenhagen, Denmark) and a Teflon®-made customized cell for SPEs, supplied by DropSens (Oviedo, Spain). Disposable Bi_SP_SPEs, consisting of a three-electrode strips configuration printed over an alumina plate surface, were provided by Dropsens. The working electrode is a sputtered thin bismuth film of 4 mm of diameter (ref. Bi10, DS SPE), the counter electrode is made of carbon and the silver pseudoreference electrode and the rest of the connections are made of silver. SPE electrodes were placed in a Teflon conic cell CFLWCL-CONIC-TEF (DropSens, Spain). The capacity of this novel cell is up to 2.0 mL sample solution for batch analysis, allowing convenient overhead stirring and spiking of the solution to perform standard addition methods. The electrodes were firmly connected to the potentiostat through a handy-modified crocodile-connector wire, in replacement of the original sliding connector that we have observed to be very prone to unexpected disconnections. All this experimental setup (Figure 1), is powered by batteries and portable, which makes it fully capable for on-site measurements. The whole system can be deployed in the field within minutes.

A standard ICP-MS protocol for Cd(II) and Pb(II) determination on a Perkin Elmer ELAN 9000 equipment (Waltham, MA, USA) was used for accuracy check of the electrochemical results.

Surface morphology characterization (image and Energy Dispersive X-Ray Analysis) was obtained by a Scanning Electron Microscope FE-SEM Quanta 3D FEG (FEI Company, Hilsboro, OR, USA). Quantitative microanalysis of Bi_SP_SPE by Time of Flight Secondary Ions Mass Spectrometer (TOF-SIMS) was carried out by a TOF-SIMS^5^ instrument (IONTOF, Munster, Germany).

### 2.3. Samples

Cd(II) and Pb(II) levels were measured in 3 different types of water: Drinking water samples, collected in urban locations (Extremadura, Spain); fortified rainwater and surface water, both collected in Badajoz (Extremadura, Spain). Drinking and raining water was properly acidified to pH 2 with HCl solution and stored at 4 °C in a fridge until analysis. The drinking water sample was analyzed on-site to demonstrate the portable features of the system.

### 2.4. Protection of the Working Electrode with Nafion

1.5 μL of a 2% Nafion solution in ethanol was carefully applied over the surface of the Bi_SP_SPEs working electrode, spreading the liquid onto the surface by the micropipette tip to obtain a homogeneous layer. The modifier was left to dry in the open air at room temperature for five minutes for complete evaporation of the solvent before use.

### 2.5. SWASV Measurements

The electrochemical measurements were made by Square-Wave Anodic Stripping Voltammetry (SWASV). Before analysis, the samples were adjusted to 0.05 M pH 4.4 acetate buffer and 4·10^−2^ M KCl. 1.5 mL of sample in the measuring cell under the following conditions: 15 s of conditioning time at −0.4, 120 s of deposition time at −1.2 V with 600 r.p.m. stirring rate, 12 s of equilibrium time at −1.2 V, potential sweep ranging from −1.2 to −0.45 V. The step potential was established at 15 mV, the amplitude at 25 mV and the frequency at 25 Hz. Finally, and after each measurement, the electrode was kept for 10 s at a potential of −0.4 V in order to clean the surface of the working electrode. It should be noted that, because the Bi_SP_SPE is not affected by the presence of dissolved oxygen, it was not necessary to deaerate the samples.

## 3. Results and Discussion

### 3.1. Influence of pH

**Figure 2:**
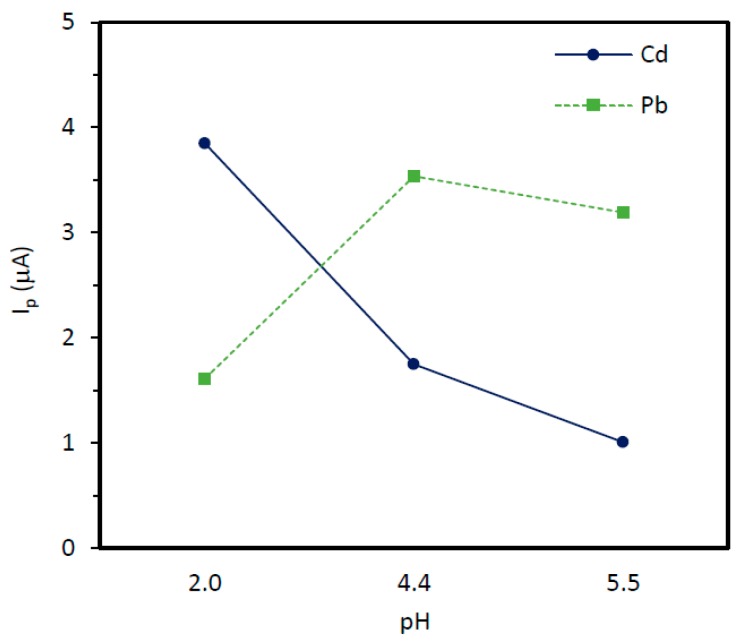
Peak current evolution of 30 ng/mL Cd(II) and 30 ng/mL Pb at the Bi_SP_SPE at different pHs. Experimental conditions: Deposition time: 120 s; deposition potential: −1.2 V; stirring rate: 600 r.p.m.; cleaning step: 15 s at −0.45 V; SWV settings: Step potential: 15 mV; frequency: 25 Hz; amplitude 25 mV; initial potential: −1.2 V and final potential −0.45 V.

According to published results, the pH values to work under optimal conditions for the joint determination of Cd(II) and Pb(II) on commercial Bi_SP_SPEs range from 2.0 to 6.0 [17,18,19]. Higher pH values are connected with a decrease in the currents and instability of the signals. In general, for voltammetric measurements, Cd(II) peak intensity increases at low pH values, and an opposite effect is observed for Pb(II) [31]. We tested the SWASV response of Cd(II) and Pb(II) on the Bi_SP_SPE in three mediums ranging from pH 2.0 to pH 5.5, and the results are shown in Figure 2. In the case of Cd(II), peak heights tend to decrease with the increase of the pH, whereas Pb(II) reaches a maximum at pH 4.4 fixed by 0,05 M acetate buffer. According to these results, pH 4.4 seems to be a good compromise for getting the best combined response from both Cd and Pb signals, so this medium was selected as the optimal value for further experiments and real samples analysis. This optimum pH could be shift to lower values if the analytical interest is focused on Cd in a particular sample.

### 3.2. Influence of Protection of the Working Electrode with Nafion

Kokkinos et al. [29] studied the influence of different Nafion solutions on bismuth film electrodes and evaluated the peak intensity for the different Nafion thicknesses resulting from each solution. They found that as the thickness of the layer increased, higher peak intensities were obtained for both metals. With this in mind, we decided to explore the influence of different concentrations of Nafion, finding similar results to those obtained by Kokkinos et al, i.e., an increase in peak intensity as the concentration of Nafion increases. However, we also found a sort of memory effect that produces an increase in the signal for the subsequent measures, using concentrations of Nafion higher than 2% and that notably increase the conditioning time between measurements. For this reason, we decided to adopt the concentration of 2% as a compromise situation between both phenomena.

**Figure 3:**
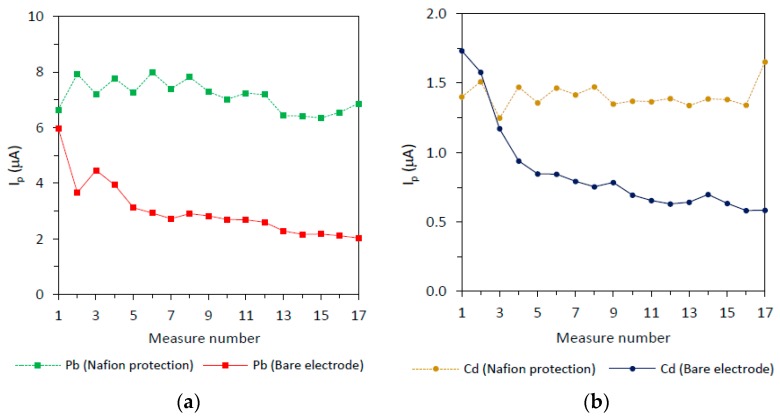
Peak current evolution for the different measure number of 30 ng/mL Cd(II) and 30 ng/mL Pb(II) at the Bi_SP_SPE with and without Nafion protection on the working electrode. Experimental conditions: Deposition time: 120 s; deposition potential: −1.2 V; stirring rate: 600 r.p.m.; cleaning step: 15s at −0.45 V; SWV settings: Step potential: 15 mV; frequency: 25Hz; amplitude 25 mV; initial potential: −1.2 V and final potential −0.45 V.

Figure 3 shows the peak intensity for the SWASV measurement of Cd(II) and Pb(II) ions on Bi_SP_SPEs with and without Nafion (three modified electrode averages) for 17 measurements. Peak intensities obtained on SPEs are typically affected by the number of measurements, generally with a slight decreasing signal trend. Moreover, the overall behavior varies slightly from one electrode to another. The somewhat delicate surface is subjected to a constant change depending on the number of measurements, being also strongly dependent on the manufacturing process of the electrodes and the subsequent handling. The signal decrease is more pronounced for the first measurements, being common or strongly recommended to discard the first measurements until achieving some stability. Moreover, although this stability could be achieved, the stable signals obtained are remarkably low affecting the detection limits.

The results for Pb(II) can be observed in Figure 3 (left). The bare electrode (solid line) shows a fast decrease at the beginning of the experiment, and then the signal even continues decreasing with a lower slope until achieving some stability after the fifth run. On the contrary, the Nafion-protected electrode (dashed line), signals are higher (2.4 times on the average) and show a much better stability during repeated measurements. The case of Cd(II) is shown in Figure 3 (right). The overall behavior is similar to that observed for Pb(II), but the declining of the signals on the bare electrode (solid line) is much more pronounced during the first analytical runs. The stability of the signals on the Nafion-protected electrode is much improved compared to the Nafion-free electrode. The peak height of Cd(II) signals on the Nafion-protected electrode is also higher than the signals measured on the bare electrode (1.6 times on the average).

**Figure 4:**
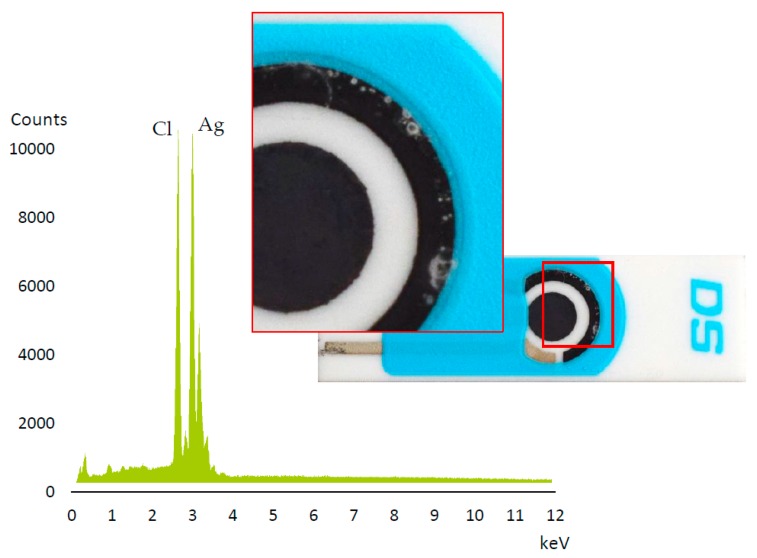
Commercial Bi_SP_SPE after 25 voltammetric runs (SWASV as indicated in Section 2.5) and EDX results.

A deposition of a white precipitate was observed on the carbon auxiliary electrode during signal stability experiments on bare and Nafion protected electrodes (Figure 4). EDX analysis of the white spots showed that it is made of AgCl, probably formed by the interaction between the silver atoms of the reference electrode and the chloride ions from the medium (mostly derived from the addition of the supporting electrolyte). This phenomenon has not been previously described in the literature about screen-printed electrodes, so it probably merits additional investigation in future work.

### 3.3. Surface Characterization of Bare and Nafion-protected Working Electrodes

As discussed in the previous section, Nafion protection may have a positive influence on electrode surface behavior and resistance to change during sequential SWASV runs, so we explore in detail the surface of the working electrode of the commercial BI_SP_SPE with and without Nafion modification, both new and after 25 SWASV runs, by applying Scanning Electron Microscopy, SEM, Time Of Flight Secondary Ions Mass Spectrometer (TOF-SIMS) and Energy Dispersive X-Ray (EDX).

**Figure 5:**
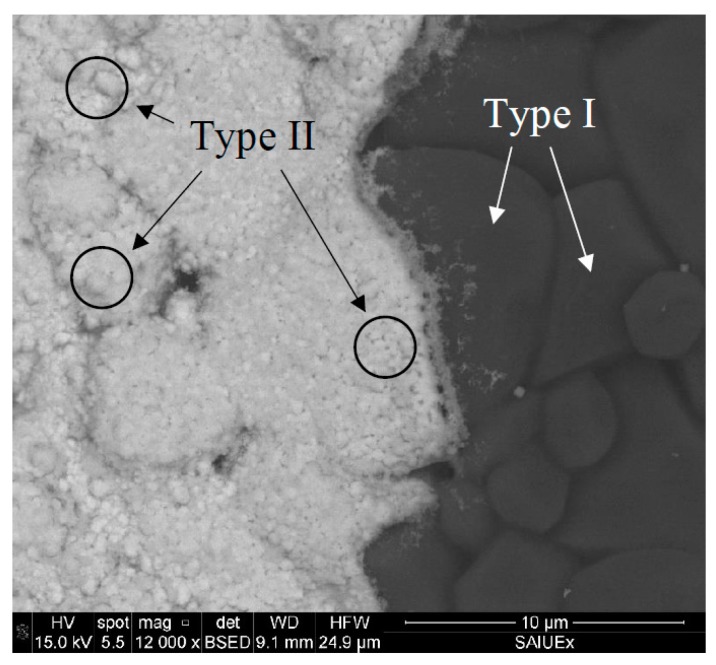
SEM image of a commercial Bi_SP_SPE: The boundary between the sputtered bismuth covering the working electrode and the bare supporting alumina substrate.

**Figure 6:**
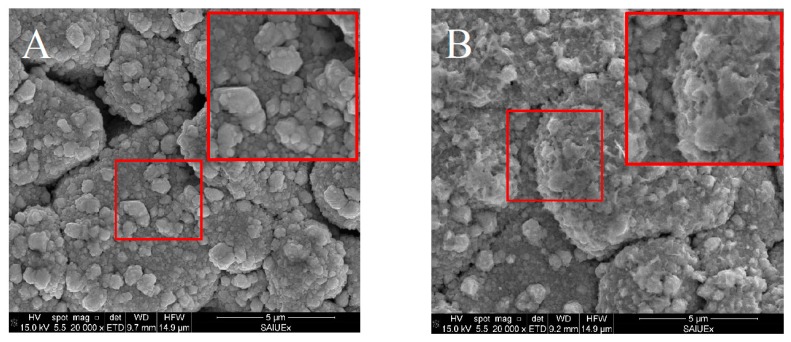
SEM images of commercial Bi_SP_SPE: (**A**) New electrode. (**B**) Same electrode after 25 voltammetric runs (SWASV as indicated in Section 2.5).

**Figure 7:**
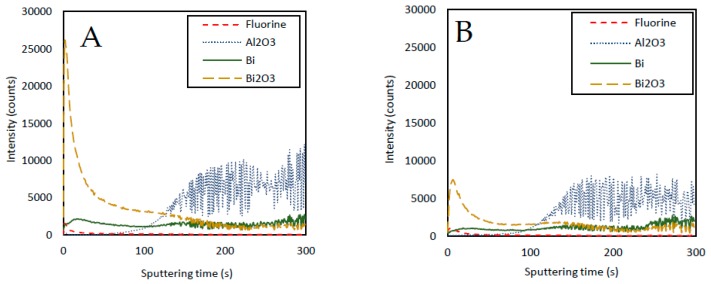
TOF-SIMS results for commercial Bi_SP_SPE: (**A**) New electrode. (**B**) Electrode after 25 voltammetric runs (SWASV as indicated in Section 2.5).

We first describe the behavior of the unprotected electrode. The superficial images obtained by SEM are shown in Figure 5, Figure 6 and Figure 7. The Bi_SP_SPE are characterized by a surface with a granular structure (Figure 5) with approximately 600–700 nm thick Bi layer [17,18]. In this structure, two types of grains can be observed: (I) Larger grains forming the supporting alumina structure (right side of the image) covered by a metallic bismuth layer (left side of the image) which are responsible for the cracks that are observed all over the surface; (II) Smaller grains formed over type I grains, which are composed of metallic bismuth.

Part of the evolution in the signal during repetitive runs (discussed in Section 3.2) could be explained by the modifications suffered by the type II structure as the number of measurements increases. As shown in Figure 6B, the surface of the electrode shown in Figure 6A has largely lost their type II structure after applying 25 measurements. This evolution could explain the smooth decreasing of the signal observed during the repetitive runs. However, to investigate the reasons for the fast downfall of the first measurements of Cd(II) and Pb(II) signals (Figure 3, solid lines) we decided to employ TOF-SIMS. This technique measures the ease of the atoms under study to detach from the surface when they are collided with impacting ions while, at the same time, it is deepened in the layers by a beam of Cs ions. Figure 7 shows how, for the first layers, a large number of Bi_2_O_3_ counts are obtained and how the counts decrease strongly as the layer is deepened. These counts are stabilized when approximately one third of the total thickness of the Bi layer is reached (approximately 125 s of sputtering). Nevertheless, the counts for metallic Bi atoms follow a constant evolution. Therefore, this Bi_2_O_3_ could be responsible for the fast signal downfall mentioned before.

**Figure 8:**
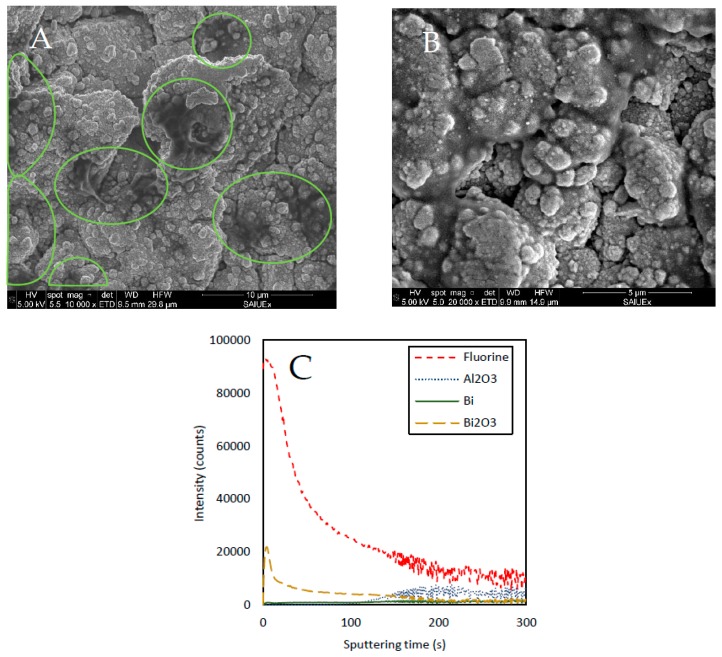
SEM image of commercial Bi_SP_SPE with Nafion additive: (**A**) New electrode modified with Nafion, (**B**) Modified electrode after 25 SWASV measurements, (**C**) TOF-SIMS results.

About Nafion protected electrodes, Figure 8 shows the SEM images obtained from a Bi_SP_SPE electrode just after the Nafion modification (Figure 8A) and a Nafion modified electrode after 25 measurements (Figure 8B). Figure 8A shows how there are certain areas where the Nafion covers part of the grains that make up the surface. It should be noted that this additive, which is not very conductive, makes difficult the visualization by SEM so, it was necessary to take high-vacuum images in order to detect at least those areas in which it was most concentrated (circled areas in Figure 8A). However, this material, although partially invisible to the microscope, is distributed with some homogeneity throughout the surface of the electrode as can be seen from the results obtained by the TOF-SIMS for a Nafion modified electrode before use (Figure 8C). These results include the study of a surface of approximately 400 × 400 μm and shows how the intensity of Fluorine, the predominant atom in the Nafion polymer, increases considerably with respect to the unprotected electrode (Figure 7A). Figure 8B shows that the protection with Nafion preserves the type II structure (smaller grains) better, after the measurements compared to a bare electrode (Figure 6B).

### 3.4. Calibration Data

**Figure 9:**
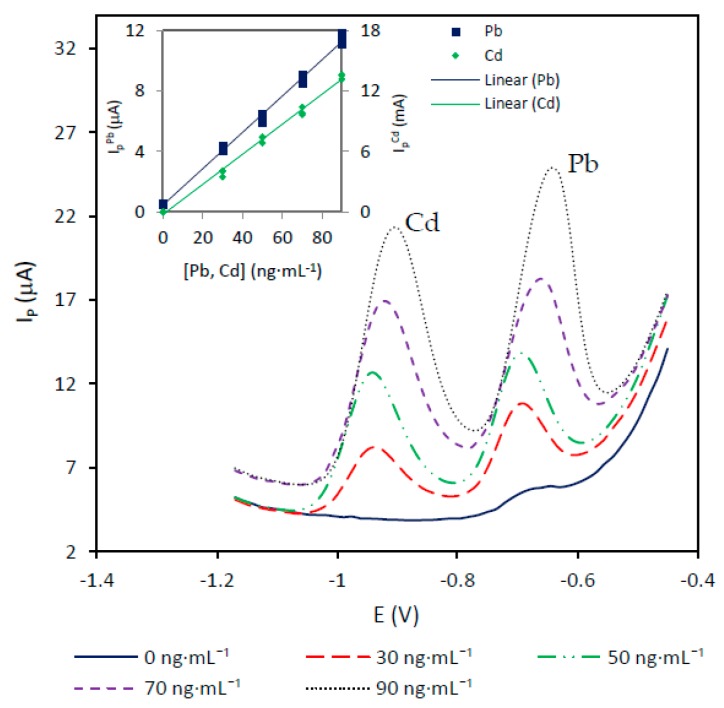
Calibration for 30–90 ng·mL^−1^ Cd(II) and Pb(II) solutions on the Nafion-protected Bi_SP_SPE in 0.05 M pH 4.4 acetate buffer. Experimental conditions: Deposition time: 120 s; deposition potential: −1.2 V; stirring rate: 600 r.p.m.; cleaning step: 15 s at −0.45 V; SWV settings: Step potential: 15 mV; frequency: 25 Hz; amplitude 25 mV; initial potential: −1.2 V and final potential −0.45 V.

To obtain Cd(II) and Pb(II) calibration data on the Nafion-protected Bi_SP_SPE, standard solutions containing both ions were analyzed in triplicate, with concentrations ranging from 30 to 90 ng·mL^−1^. Figure 9 shows the voltammograms obtained and the calibration curve using 0.05 M pH 4.4 acetate buffer as a medium. Table 1 summarizes the main parameters of the calibration. As can be seen in the table, the detection limits, calculated according to Long and Winefordner, are 3.62 and 3.83 ng·mL^−1^ for Cd and Pb respectively. Thes detection limits are better than those reported by Palomo-Marín et al. [17], for an application of the Bi_SP_SPE to the voltammetric measurement of Cd and Pb in atmospheric aerosols using 0.1 M HCl (pH 1) medium. However, the detection limits obtained on the Bi_SP_SPE protected with Nafion are higher than those reported by Sosa et al. [18,19] for the application of an unprotected Bi_SP_SPE to Cd and Pb determination in natural waters. These authors used conventional auxiliary and reference electrodes in addition to the working Bi_SP_SPE to complete the electrochemical cell. Lower detection limits were also reported by Kokkinos et al. [29] using a non-commercial Nafion-modified microfabricated bismuth microelectrode (0.5 ng·mL^−1^ for both Cd and Pb).

To estimate the repeatability of the Nafion-protected Bi_SP_SPE, 10 voltammetric measurements of a solution containing 30 ng·mL^−1^ Cd(II) and Pb(II) were carried out on a single Bi_SP_SPE. Relative standard deviation (RSD) was 6 and 7 % for Pb(II) and Cd(II) respectively. The reproducibility of electrode to electrode measurements was also evaluated using a set of 3 different electrodes, giving RSD values of 34 and 17 % for Pb(II) and Cd(II) respectively. Values for repeatability are acceptable taking into account the disposable characteristics of these low cost electrodes. However, RSD values for reportability are relatively high, making it necessary to carry out calibration for each electrode before using them for measurements in real samples.

### 3.5. Analysis of Real Samples

The samples of natural water selected for the evaluation of the applicability of the method to on site analysis of real samples were analyzed in triplicate. The results obtained by SWASV were compared with a standard ICP-MS protocol. Each electrode was calibrated with three Pb(II) and Cd(II) standard solutions before measuring the real samples, for quantification by external calibration. Table 2 shows Cd(II) and Pb(II) concentrations obtained for the three types of samples of natural water. These samples contain exceeding amounts of potentially interfering ions as Zn(II) and Cu(II). As can be seen, the SWASV results are in agreement (recoveries from 75 to 111%) with those obtained by the ICP-MS reference method. It should be noted that the value obtained for the Pb(II) in the drinking water sample is above the legal limit, due to the fact that this sample was taken from a facility using lead pipes. The Nafion-protected Bi_SP_SPE allowed for proper monitoring of Pb(II) for discrimination between samples above legal limits that therefore poses a risk to human health.

## 4. Conclusions

The protection of the screen-printed sputtered bismuth electrode (BispSPE) with a layer of Nafion significantly improves its analytical performance for the determination of Cd and Pb by means of square wave anodic stripping voltammetry, increasing the magnitude of the signal 2.4 times for Pb and 1.6 times for Cd. Nafion protection also significantly improves the stability of the electrode response against repeated measurements, allowing a complete calibration cycle plus repeated measurements of real samples on the same electrode. The detailed exploration of the morphology of the electrodes by microscopy and superficial analysis techniques has shown that the protection with Nafion allows better conservation of the original structure of the electrode during repeated measurements processes, which is probably the reason for its better performance. The optimization of chemical and instrumental parameters has allowed the calibration of the method on the Nafion modified electrode, with enough detection limits for the measurement of the concentration of Cd and Pb in samples of natural waters. The electrode has been used in a miniaturized cell, in combination with a battery-operated stirring system, portable potentiostat, and laptop, to generate a completely portable analytical system, suitable for analysis on site. The applicability of the method has been demonstrated for decentralized measures in drinking water, rainwater, and surface waters, with recoveries between 75% and 111% with respect to an ICP-MS reference method.

## Figures and Tables

**Table 1:** Calibration data for the determination of Cd(II) and Pb(II) on a Bi_SP_SPE in 0.05 M pH 4.4 acetate buffer.

Metal Ion	m	b	S_m_	S_b_	S_y_/X	AS (ng·mL^−1^)	R^2^	L (%)	LOD (ng·mL^−1^)
Cd(II)	0.149	−0.274	0.003	0.180	0.381	2.55	0.994	97.89	3.62
Pb(II)	0.120	0.438	0.003	0.147	0.311	2.59	0.994	97.86	3.83

AS: Analytical Sensitivity, L: Linearity. LOD: Limit of Detection, m: Sensitivity (μA/ng·mL^−1^).

**Table 2:** Cd(II) and Pb(II) in real samples. 95 % confidence intervals in brackets.

Sample	Cd SWASV (ng·mL^−1^)	Cd ICP-MS (ng·mL^−1^)	Pb SWASV (ng·mL^−1^)	Pb ICP-MS (ng·mL^−1^)
Drinking water	< LOD	< LOD	93.28 (1.59)	83.92 (1.82)
Fortified rainwater	47.59 (1.33)	57.82 (1.92)	43.25 (1.79)	52.03 (0.18)
Fortified surface water	32.34 (1.11)	43.13 (0.64)	33.59 (4.23)	43.47 (0.76)
